# The Non-Hemostatic Aspects of Transfused Platelets

**DOI:** 10.3389/fmed.2018.00042

**Published:** 2018-02-27

**Authors:** Caroline Sut, Sofiane Tariket, Cécile Aubron, Chaker Aloui, Hind Hamzeh-Cognasse, Philippe Berthelot, Sandrine Laradi, Andreas Greinacher, Olivier Garraud, Fabrice Cognasse

**Affiliations:** ^1^GIMAP-EA3064, Université de Lyon, Saint-Étienne, France; ^2^Etablissement Français du Sang, Auvergne-Rhône-Alpes, Saint-Etienne, France; ^3^Médecine Intensive Réanimation, Centre Hospitalier Régionale et Universitaire de Brest, Université de Bretagne Occidentale, Brest, France; ^4^Institute for Immunology and Transfusion Medicine, University of Greifswald, Greifswald, Germany; ^5^Institut National de Transfusion Sanguine (INTS), Paris, France

**Keywords:** platelets, transfusion, CD40L, serious adverse reaction, inflammation, innate immunity

## Abstract

Platelets transfusion is a safe process, but during or after the process, the recipient may experience an adverse reaction and occasionally a serious adverse reaction (SAR). In this review, we focus on the inflammatory potential of platelet components (PCs) and their involvement in SARs. Recent evidence has highlighted a central role for platelets in the host inflammatory and immune responses. Blood platelets are involved in inflammation and various other aspects of innate immunity through the release of a plethora of immunomodulatory cytokines, chemokines, and associated molecules, collectively termed biological response modifiers that behave like ligands for endothelial and leukocyte receptors and for platelets themselves. The involvement of PCs in SARs—particularly on a critically ill patient’s context—could be related, at least in part, to the inflammatory functions of platelets, acquired during storage lesions. Moreover, we focus on causal link between platelet activation and immune-mediated disorders (transfusion-associated immunomodulation, platelets, polyanions, and bacterial defense and alloimmunization). This is linked to the platelets’ propensity to be activated even in the absence of deliberate stimuli and to the occurrence of time-dependent storage lesions.

## Introduction

**Figure 1:**
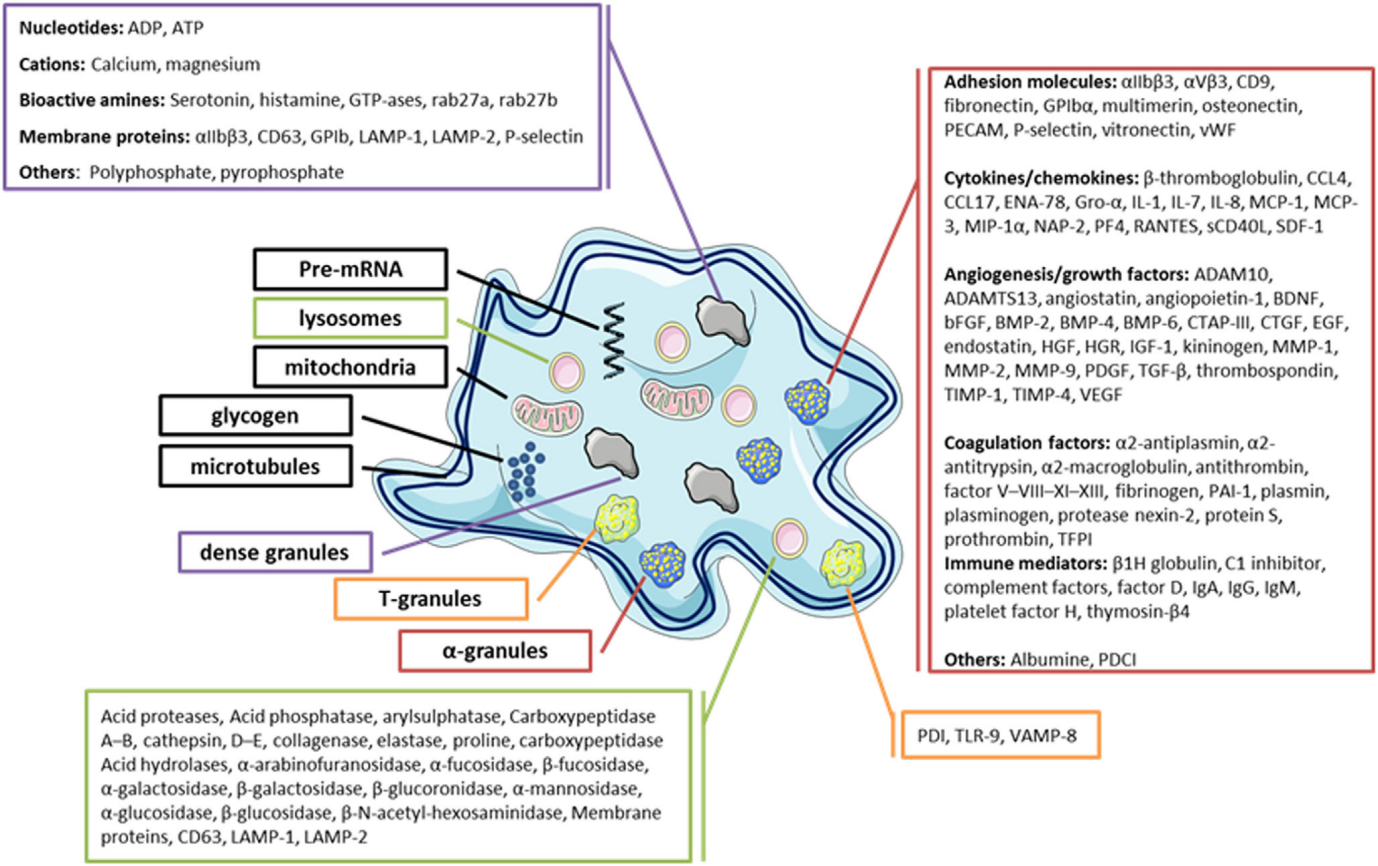
Diagram of the platelets’ main granules and their secretory content. Most products that can be released by platelets are listed.

Blood platelets are small anucleate cells essentially originating from megakaryocyte (MK) fragmentation. These cells have a dense cytoskeleton that maintains their discoid shape in normal state and changes the platelets to a spherical form after their activation (1). Platelets play a key role in vascular repair and maintenance of homeostasis, particularly in primary hemostasis. The platelet membrane glycoproteins can interact with the elements of the injured endothelium, mediating their adhesion, followed by activation and finally aggregation, resulting in the formation of a thrombus formed by aggregation of interconnected platelets by fibrinogen to close the vascular gap (1, 2). Platelets also play an important role in innate and adaptive immunity by interacting directly or indirectly with other immune cells to trigger or maintain the inflammatory response (1–3). Several factors are involved in the platelet inflammatory process, in particular, by membrane expression of several immune receptors, such as cytokines (CKs), chemokines (CHs), and a large number of soluble factors contained in their granules (in α-granules, this includes CKs/CHs, immunomodulatory factors, and growth factors, etc.) (4, 5) (Figure 1). Moreover, platelets also release other factors: (i) growth factors promoting angiogenesis, which are also required to repair damage to inflammatory sites (6–8), (ii) clotting factors required for platelet hemostatic functions (9, 10), (iii) antibacterial peptides (1, 11), (iv) adhesion factors (12), and (v) inflammatory mediators, such as serotonin and histamine (13–15).

Platelet-derived soluble CD40L (sCD40L) is a key mediator of the immune system (16–19). Platelet receptor and signaling is important and drive the stimulated platelet granule secretion in these differential profiles—a completely new concept with regard to an anucleate cell—also appear to be strictly regulated by intraplatelet signaling pathways, depending on the stimuli (20–23). This review summarizes current information surrounding the association between inflammation and transfused platelets.

## A Brief Overview of Platelet Functions

Blood platelets are important reservoirs of soluble, preformed mediators (CKs/CHs, hemostatic factors, and immunomodulators) that are present in secretory granules, in particular, α-granules, δ-granules, and lysosomes, which are released upon their activation (1, 15, 24–28). Platelets contain a large variety of CKs/CHs, which are mainly synthesized in the MK and stored in α-granules in most cases (Figure 1). CKs/CHs may directly interact with cells of the innate and adaptive immune system or indirectly through immune or non-immune relay cells, such as endothelial cells. CK/CH platelets help regulate the surrounding cells, including their proliferation, differentiation, and activation (1). Interestingly, platelets also express receptors for several CKs/CHs that they secrete, showing their potential to establish autocrine and paracrine bidirectional loops. Platelet immunomodulatory factors include growth factors and CKs/CHs, but also molecules sharing the main characteristics of CHs and CKs, such as sCD40L/CD40L, soluble P-selectin/CD62P, platelet-derived growth factor AB (PDGF-AB), transforming growth factor β, interleukine 1β, regulated on activation normal T cell expressed and secreted (RANTES), and platelet factor 4 (PF4 ou CXCL4).

Although platelets have generally not been considered central to innate immunity and inflammation, this paper provides evidence that platelets may play a key role. Recent data report evidence that platelets can also recycle a number of CKs/CHs and regulatory products called biological response modifiers (BRMs) for which they also express the pairing ligand, which is the case for sCD40L (platelets are the major purveyors of this molecule in the circulation) (17, 29). Platelets also express membrane CD40 and, unlike CD40L, CD40 is detectable on the surface of resting as well as activated platelets (1).

Cytokines and other platelet products can be readily detectable at the onset of acute inflammation (8). In this regard, transfusion is an excellent model of the pathological process, as here, the mediators of inflammation are transfused with consecutively rare, but then, severe serious adverse reaction (SARs). It is currently and widely admitted that sCD40L is the master platelet-associated CKs (17, 30). When it was first described in 2001 in association with platelets, this was in the context of platelet component (PC) transfusion hazards. Subsequently, platelet-sCD40L and the CD40/CD40L pair have been described in many pathologies. The conclusion that, e.g., febrile non hemolytic transfusion reaction (FNHTRs), where sCD40L appears to be chiefly responsible for pathological symptoms, was indeed inflammatory. This conclusion seems similar to their role in diabetes, cardiovascular disease, atheromatous plaques, and inflammatory bowel disease, where CD40/CD40L have now been acknowledged as being influential (1, 31).

## Platelet Interactions with Other Blood Cell Elements

Interactions of sCD40L with its CD40 receptor (expressed on immune cells or other cells, such as endothelial cells) can modulate the responses of each of the different cell partners (5, 32). Indeed, platelet sCD40L, interacting with CD40 on endothelial cells, induces inflammatory responses characterized by the expression of adhesion receptors (E-selectin, P-selectin, intercellular adhesion molecule 1, vascular cell adhesion molecule 1) for the release of proinflammatory CKs/CHs (CCL2, IL-6, IL-8) and the recruitment of leukocytes to the inflammatory sites (16). The *in vitro* engagement of neutrophil CD40 by sCD40L induces the generation of reactive oxygen species (ROS) and the destruction of lung endothelial cells suggesting this factor’s role in transfusion-related acute lung injury (TRALI) (33). Moreover, platelet sCD40L creates a link between innate and adaptive immunity in promoting maturation (19, 34), activation (35), secretion (36), and presentation of antigen by dendritic cells (DCs), which are cells capable of activating naive T cells to induce an adaptive immune response (5). Elzey et al. have demonstrated that platelet sCD40L can, both *in vitro* and *in vivo*, amplify the activity of pathogen-specific CD8+ T lymphocytes (Lyt), which results in the production and function of IFNγ, and in the enhancement of their lytic function (37). Iannacone has further shown that the number of cytotoxic Lyt during infection with lymphocytic choriomeningitis virus was dramatically reduced in the absence of platelets, involving CD40/CD40L: thrombocytopenia is estimated to result almost exclusively from the antiplatelet antibodies (38). CD40/CD40L also plays a major role in the interaction of CD4^+^ T lymphocytes (and CD8+) and B lymphocytes, which supports proliferation, differentiation, and production of immunoglobulin by plasma cells. Platelet or MK-derived sCD40L, which is continuously released into the circulation in large quantities (37, 39, 40), is a key molecule regulating the immune system and increased release of sCD40L plays a major role in the pathogenesis of the immune-mediated disease.

Platelets are also essential for the formation of neutrophil extracellular traps (NETs) by neutrophils, The NET formation is an apoptotic process, most important to release of neutrophil DNA, which entraps bacteria resulting in bacterial clearance and concentrating antibacterial factors but in enhancing thrombosis. Toll-like receptor 4-activated platelets bind to neutrophils and initiate NET formation. Platelets facilitate NETosis *via* several protein interaction as CD62P-PSGL-1, involving of platelet GPIbα or neutrophil lymphocyte-function-associated-antigen-1. Moreover, platelet release several soluble factor initiate NET formation and increase bacterial clearance [CXCL4, von Willebrand factor, high-mobility group box 1 protein, thromboxane A2, and β-defensin (41)]. Platelet–leukocyte interactions has focused on platelet interactions with monocytes and neutrophils, as described above, but platelets present a role in T cell responses. Chapman et al. show elegantly that platelets express T cell costimulatory molecules, process, and present Ag in MHC class I and directly activate naive T cells in a platelet MHC class I-dependent manner. The group of Craig N. Morrell define new concept that platelets not only support and promote acquired immune responses but platelets may also directly participate in the initiation of acquired immune responses (42). While for the role in primary hemostasis, platelets primarily interact with endothelial cells, they also interact directly or indirectly *via* their released CK/CH with many of cell types, hereby, strongly influence their function. Platelets can, indeed, activate (and be mutually activated by) almost all types of leukocytes (monocytes, T-lymphocytes, B-lymphocytes, and neutrophils) and DCs (1, 30, 43). When allogeneic (donor) platelets are transfused to patients, the recipients’ circulating cells make foreign encounters [e.g., by human leukocyte antigen (HLA) class I molecule expressed on platelets] and can potentially be activated by those encounters, and vice versa. This led to a recent re-examination of the concept of pathogens defense mechanisms, extending it to non-infectious “dangers” such as foreign (transfused) cells (15, 26, 27, 44, 45). PCs are stored for a maximum of 5 days (most countries) before being issued to a patient in need; prior to that, during their shelf life, platelets “spontaneously,” i.e., with no acknowledged exogeneous stimulus, release a number of CKs, particularly sCD40L (17, 30) in high enough quantities to exert functional activities on target cells possessing the *ad hoc* receptors. sCD40L was found to be consistently and significantly elevated in PCs that had led to SARs comprising various syndromes, including (antibody independent) TRALI (although this is disputed in such particular case) (30, 33).

## A Brief Overview of PC Transfusion Benefits and Complications

Platelet component transfusions have two main indications, aimed at being either curative or prophylactic (46). Curative transfusions are given to patients presenting with active bleeding and low to very low platelet counts (in exceptional circumstances, the platelet count can be normal, but platelets are non-functional), or massive blood loss. Curative transfusions are not under debate, unlike the protocols and timing of other blood component transfusions [red blood cell concentrates (RBCCs) and fresh plasma] and/or blood derivatives, such as prothrombin complex concentrate or fibrinogen. There is no consensus on prophylactic transfusions, however, although many practitioners still recommend not exposing at-risk patients to bleed. Thresholds for transfusion and quantities of transfused platelets vary consistently in different countries and with different systems. In short, PC transfusion provides a benefit to patients and prevents bleeding and deterioration of otherwise serious clinical conditions. PC transfusion is supportive in many chemotherapy protocols and stem cell transplantation.

Platelet component transfusions can lead to adverse inflammatory reactions. The majority of adverse inflammatory reactions in patients receiving blood (recipients) appear either FNHTRs or allergy, both being clearly inflammatory conditions. FNHTR is characterized and associated with fever (≥38°C or ≥1°C above baseline, if baseline ≥37°C), or chills and rigors, but not directly with hemolysis, caused by cytokines that accumulate in the product during storage. FNHTR is also initiated by the presence of recipient antibodies reacting to donor HLA or other antigens. Allergic reactions (e.g., urticaria) occur within minutes after the start of the transfusion. Allergic reactions may be associated with mild upper respiratory symptoms, nausea, vomiting, abdominal cramps, or diarrhea. Allergic reactions could be severe (e.g., anaphylaxis). Patients can present a severe hypotension, cough, bronchospasm (respiratory distress and wheezing), laryngospasm, angioedema, urticaria, nausea, abdominal cramps, vomiting, diarrhea, shock, and/or loss of consciousness. This may be a fatal reaction. Severe allergic reactions could be dependent of (i) IgA-deficient patients who have anti-IgA antibodies, (ii) patient antibodies to plasma proteins (such as IgG, albumin, haptoglobin, transferrin, C3, C4, or cytokines), (iii) transfusing an allergen to a sensitized patient (for example, penicillin or nuts consumed by a donor), or (iv) rarely the transfusion of IgE antibodies (to drugs, food, etc.) from a donor to an allergen present in the recipient (47).

On rare occasions, PC transfusion can lead to immediate to short-delayed inflammatory adverse reactions (grades 1–3: 0.24%); however, in some cases (0.006%), grade 3 reactions can be life-threatening (48). The rationale for the relatively high number of SARs with PC transfusion [from 1/4 to 1/2 of all reported SARs, while PCs represent only about 10% of transfused blood components (BCs)] may be deduced from their propensity to secrete copious amounts of pro-inflammatory BRMs as outlined in the previous section. In addition, PC transfusion can be associated with volume overload, as PCs frequently come into large volumes and elevated levels of proteins and lipids exerting a surfactant effect (49). The latter can be prevented by close patient monitoring and by replacing 2/3 of plasma with platelet additive solutions (50). The case of TRALI and the responsibility of platelets have been presented elsewhere (45). Finally, PC transfusion carries a greater risk of bacterial contamination, which can be life threatening especially in severely immuno-compromised patients (51). The introduction of Pathogen Reduction Technologies has abrogated much of the adverse effects associated with pathogen contamination of platelet products (52, 53). Pre-storage leukoreduction proved to significantly reduce inflammatory reactions as well as viral infections (54). In brief, PC transfusions can induce unwanted effects, e.g., volume, plasma, inflammatory reactions, pathogen transmission, etc., in addition to their therapeutically intended effect, i.e., improving hemostasis. However, since PC-transfused patients are particularly fragile patients, close monitoring and careful dosing can prevent many complications such as volume overload.

## Platelet Storage and Outcomes of Critically Ill Patients

**Figure 2:**
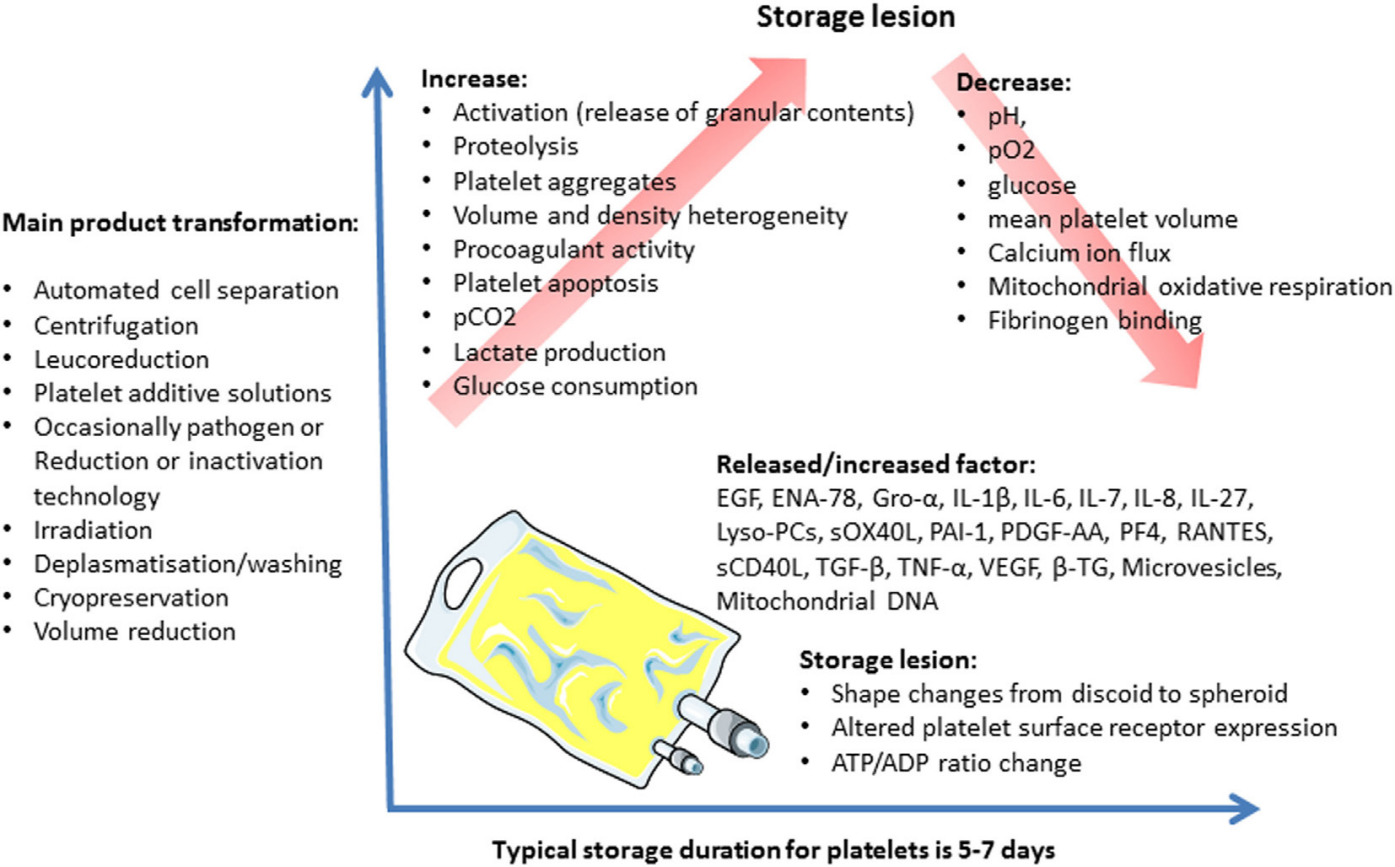
Platelet concentrate storage and biological response modifier release.

Over the platelet storage period, certain biochemical and functional changes occur in the platelets and their storage medium. These changes, called storage lesions (Figure 2), include acidification of the storage medium secondary to anaerobic platelet metabolism, platelet activation (55), and an increase in CKs and lipids level in PCs (56, 57). Several authors applied a metabolomics approach to the issue of donor variability in poststorage platelet viability. Metabolomic analysis of the stored platelets identified multiple specific metabolites that correlated with either PLT recoveries or survivals after transfusion (Lipid metabolism components, caffeine, and its metabolites) (58). Interestingly, platelet storage lesion is not associated with a linear decay of metabolism, but rather with successive metabolic shifts (59). Prudent et al. review the key findings of the proteomic analyses of platelet concentrates (PCs) treated by the Mirasol Pathogen Reduction Technology, the Intercept Blood System, and the Theraflex UV-C system, respectively, and discuss the potential impact on the biological functions of platelets. The impact of the Pathogen inactivation treatment on the proteome appears to be different among the Pathogen inactivation systems (53, 60).

These storage lesions may compromise the platelets’ viability and functionality, and, therefore, the transfusion’s efficacy (61). They may also lead to adverse reactions in the recipients.

Critically ill patients are the second largest patient group to receive platelet products after oncology–hematology patients. Around 15% of critically ill patients require a platelet transfusion during their intensive care unit stay for treatment or prophylaxis of bleeding (62). Critically ill patients are characterized by a coexisting inflammatory state, making them theoretically more susceptible to blood product adverse reactions. A “two-hit” hypothesis has largely been used to explain the pathophysiology of transfusion adverse events including TRALI, the first hit being a pro-inflammatory condition and the second hit being the administration of antibodies or BRMs through blood component transfusion (63). Results of *in vitro* and animal studies suggest platelets storage lesions have a key effect on the occurrence of non-antibody mediated TRALI (33, 63). Khan et al. have observed an increase in sCD40L level over the PC storage period, and higher levels of sCD40L in platelet products implicated in TRALI, suggesting that the accumulation of sCD40L during platelet storage induces TRALI (33). Consistent with these findings, Vlaar et al. have found that stored platelet supernatant compared with fresh platelet supernatant led to an increase in systemic and pulmonary coagulopathy in lipopolysaccharide pretreated rats (63).

CD40/CD40L complex could be a major target in a TRALI prevention strategy. Improving the conditions in which the PCs are prepared and stored would contribute to controlling partly the risks of non-immune TRALI.

Prolonged platelet storage has been associated with a decrease in posttransfusion platelet increment and a shorter time to next platelet transfusion in oncology–hematology patients (64–66), but the clinical consequences of the platelets storage lesions remain uncertain (66–68). To our knowledge, no study has investigated the association between transfusion efficacy and platelet storage duration in critically ill patients. Five observational studies have investigated the association between PC storage duration and critically ill patient outcomes; one included post-cardiac surgery patients only, two studies included trauma patients only, and two all critically ill patients (69–73). There was no association between mortality and storage duration in the three studies evaluating this outcome (69, 70, 73). In a study of 381 trauma patients, those receiving platelets stored for 5 days developed more complications, including sepsis, than patients transfused with platelets stored for less than 5 days (5.5% sepsis in patients receiving platelets stored for 3 days or less, versus 16.7% in patients receiving platelets stored for 5 days, *p* = 0.03) (70). After adjustment for confounders, patients receiving PCs stored for 5 days had a 2.4-fold higher risk of developing complications, including acute renal failure, acute respiratory distress syndrome, and sepsis, than patients transfused with fresher platelets (70). All these studies are retrospective and have numerous limitations in their methods making it impossible to draw any definitive conclusion on the impact of platelet storage duration on clinical-centered outcomes. Prospective research is warranted to determine whether prolonged platelet storage has an impact on the prognosis of critically ill patients. In the meantime, better understanding of platelet transfusion-related immunomodulation may help us to understand the reported association between platelet transfusion and an increased risk of hospital-acquired infections (74, 75).

## Transfusion-Associated Immunomodulation

Transfusion-related immunomodulation or TRIM is a complex event with dual effects that are potentially beneficial, but in general, mostly considered harmful (76). The long-term effect of transfusions is suspected to modulate (dampen) immune responses and consequently favor the emergence of secondary malignancies and infections. It is, however, extremely difficult to decipher the respective roles of causal pathologies in severely sick intensive care patients or patients receiving chemotherapy and immunosuppressants, monoclonals and biosimilars, and BCs. TRIM induced by PCs would be best understood in patients having received PCs only, but it is almost impossible to delineate the immunosuppressive role of platelets relative to red blood cells as only very few patients receive PCs and no RBCCs. Furthermore, in case of plasma-rich BCs, plasma polyreactive immunoglobulins (Igs) may counterbalance certain immunosuppressive effects. In short, whether PC transfusions may be immunomodulatory remains elusive and difficult to assess, though it would be of interest to investigate this in order to provide patients with optimized care.

## Platelets, Polyanions, and Bacterial Defense

While the above-described mechanisms clearly indicate that platelets interfere with the immune system, only a few studies clearly show a causal link between platelet activation and immune-mediated disorders. One well-investigated example of the role of platelets in mediating immune reactions is the interaction of platelets with heparin.

The adverse drug effect of heparin-induced thrombocytopenia (HIT) will, therefore, be used to exemplify the interaction of platelets and the immune system. HIT is a prothrombotic adverse drug reaction caused by the transient production of IgG-class platelet-activating antibodies that recognize multimolecular complexes of the positively charged PF4 and the polyanion drug heparin. These antibodies activate platelets and also monocytes *via* their FcγRIIa receptors. This causes transformation of an immune reaction into a prothrombotic reaction, resulting in massive thrombin generation and paradox thrombotic complications. If unrecognized, the risk for new thrombosis in affected patients is 5% per day and the risk of mortality is 25–30% (77). There is no doubt that with HIT, platelets mediate an extremely powerful reaction, which results from concomitant activation of the immune system and the coagulation system.

The reason for this massive response is that HIT is likely a misdirected bacterial host defense (78). PF4 binds charge-related to Gram-negative and Gram-positive bacteria. On Gram-negative bacteria, lipid-A is the binding site for PF4 (79). The binding site of PF4 on Gram-positive bacteria has not yet been identified. The question raised is how and why PF4 induces such a potentially dangerous immune response.

The following section summarizes our recently proposed working model (80, 81). All bacteria expose strong negative charges on their surface. This negative charge is likely a mechanism by which bacteria are kept apart from each other, and by which bacteria are protected from phagocytosis. The zeta potential-mediated repulsive forces generated by the negative charges push bacteria apart from each other and away from their “predators” (the reader is invited to watch the following YouTube video demonstrating this principle https://www.youtube.com/watch?v=Kb-m1uDoWfU). Eukaryotic cells, however, must not have this strong negative charge as the repulsive forces would be incompatible with a complex multicellular organism. In view of this consideration, we propose that a strong negative charge is a fundamental feature of prokaryotes. In line with this concept, basic mechanisms of the innate immune system, like the alternative and classic complement pathway, the intrinsic clotting system with factor XII and factor XI, as well as the kininogen–bradykinin pathway are strongly activated by negative charges (82). However, the adaptive immune system (T cell receptors, B cell receptors, antibodies) do not recognize charge, they recognize structures. The platelet-derived CH PF4 has the role of translating charge into structure. After binding to negative charges, PF4 undergoes complex structural changes [for review, see Ref. (83, 84)]. These structural changes expose a neoepitope, which is recognized by anti-PF4/polyanion antibodies, the same antibodies that induce HIT. After binding of anti-PF4/P antibodies to PF4-labeled bacteria, these opsonized bacteria mediate very efficient phagocytosis by granulocytes (78). The evolutionary advantage of using such a mechanism is that it enables an early IgG response toward bacteria the organism has not seen before. The newly encountered bacteria also bind PF4; PF4 undergoes its conformational change due to the negative charge on the bacteria surface and is then recognized by the preformed anti-PF4/P antibodies. In line with this concept, most likely a secondary immune reaction, natural anti-PF4/P antibodies are found in the general population where their presence is highly correlated with the presence of chronic infections like chronic periodontal disease (85). On the basis of this concept, these antibodies must be very common. Indeed, the adverse drug reaction HIT has helped to prove this. In HIT, anti-PF4/P IgG are formed in high titer between day 5 and day 10 (86). As B cells cannot produce IgG antibodies during a primary immune response within 5–10 days, HIT is always a secondary immune reaction, even in patients who have never received heparin before. As 65% of patients develop these antibodies after cardiac surgery, a plausible explanation for such frequent primary immunization is the above-outlined concept of bacterial infection-related priming of the immune system.

However, the above-outlined concept of the role of PF4 and platelets as mediators between innate and specific immunity places platelets in a very special position, bridging two major parts of our immune defense system. Platelets secrete or expose many molecules with a specific role in immunity. Little information exists on how platelet storage modifies the structure of these molecules or their spatial presentation within platelet compartments or on the platelet surface. As exemplified by the structural changes of CH PF4 induced by polyanions like heparin, conformational changes in these proteins may transmit a danger signal to the transfusion recipient’s immune system, which erroneously triggers potent pathogen defense mechanisms, resulting in adverse transfusion reactions. Although this has not been shown yet, it is conceivable that other platelet-derived mediators such as sCD40L intensify and probably orchestrate the interaction of platelets and other immune cells with pathogens. If misdirected, this can cause SARs. The adverse drug reaction of HIT provides one of the most prominent examples of the potentially deleterious consequences for patients.

The risk that our immune system develops autoimmune-like reactions toward platelet proteins when they are modified during storage is probably quite low, although such autoimmune reactions may occur. Again, this has been demonstrated for the immune reaction toward conformationally changed PF4. In the past decade, it has become recognized that certain patients present with clinical symptoms and laboratory features of HIT despite not having previously received heparin either in the recent past or at all. Sera from these patients contain antibodies that strongly activate platelets even in the absence of heparin. To date, ≈20 cases of spontaneous HIT syndrome have been reported (87–96). In the plasma of these patients, antibodies are found, which bind to PF4 with such high avidity that they cluster two PF4 molecules, thereby inducing the same conformational change as polyanions. These clusters of conformationally changed PF4 attach to platelets and endothelial cells, giving the immune system a false signal of the presence of strong negative charges, which prompts the above-described bacterial defense mechanism.

As the negative charge is a danger signal for the human defense system, bacteria have naturally developed counteracting methods to hide this danger signal. One of which is long lipopolysaccharide (LPS) chains covering and “hiding” the negative charges or the Fc-part of the anti-PF4/P antibodies bound to conformational-changed PF4 on the bacteria surface. Lipid A is the basis of LPS. PF4 has a diameter of 5 nm; when an IgG molecule (which is about 10 nm long) binds to conformationally changed PF4 bound to lipid A, the entire complex has a height of about 15–18 nm. The LPS chain, however, can reach lengths of up to 25 nm. This covers the Fc part of the antibody and thereby recognition of opsonized bacteria by the immune system’s Fc receptors. However, platelets, in addition to PF4, secrete polyphosphates from their δ-granules. Polyphosphates are also negatively charged and bind to the PF4 molecule on the bacteria surface, attracting other PF4 molecules and finally forming large multimolecular PF4/polyphosphate complexes, which extend well out of the bacteria’s LPS shield (97). This has two effects: conformationally changed PF4 is now exposed for antibody recognition, and consequently several anti-PF4/P antibodies can bind to these complexes on the bacterial surface, forming immune complexes, which are then readily recognized by the Fc receptors of human defense cells.

Platelets also found a new way in which platelets defend against bacteria. When platelets are incubated with *Escherchia coli* in the presence of PF4 and anti-PF4/P antibodies, platelets kill up to 75% of *E. coli* by direct platelet bacteria interaction. Upon investigating this mechanism in more detail, we found a new way in which platelets defend bacteria. It is well established that platelets can internalize IgG-coated targets (98–100); however, it is debated whether phagocytosis of bacteria (i.e., *Staphylococcus aureus*) (101) is really a major mechanism for bacterial host defense (102, 103). Although platelets store bactericidal substances in their α-granules (12), α-granules are designed to be released and it is unlikely that phagocytosed bacteria are transported within the platelet into the α-granule. Such a mechanism would be incompatible with platelet shape change during activation where platelets are spread thinly over a large area with the α-granules concentrated within the immediate granulomere zone (104). We propose an alternative mechanism, where platelets cover bacteria by widely extending their membranes and then actively contracting them, thereby centralizing bacteria until they are very close to the granulomere of the platelets, where the substances with antibacterial potency are stored (105). When a threshold concentration of platelet-activating signals is reached due to platelet interaction with the opsonized bacteria, the activated platelets release their α-granules preferentially at the site of the bacteria, thereby locally reaching high concentrations of antibacterial substances. This phenomenon is similar to the pore-forming perforin released from the granules at the immunological synapse potentiated by cytotoxic T lymphocytes (106).

The above-outlined mechanisms are not the only ways in which platelets interfere with bacteria and other pathogens (107–110). Through complex mechanisms involving the platelet Fc-receptor FcγRIIA (111–113), glycoprotein αIIbβ3, GPIbα, complement receptors (e.g., gC1q-R), and toll-like receptors (e.g., TLR-2 and TLR-4), platelets interact with bacteria and become activated by bacteria (27, 114). Upon activation, platelets release antimicrobial substances such as ROS, antimicrobial peptides, defensins, kinocidins, and proteases (11, 115–117). Taken together, there is ample evidence that platelets play an important role in the defense against pathogens.

Recognition of pathogens by platelets is at least partly mediated by conformationally changed endogenous, platelet-derived proteins. The challenge for transfusion medicine and immunohematology is to identify whether platelet proteins with an important role in danger signaling are also conformationally changed during platelet processing and storage, thereby presenting a danger signal with an increased risk of triggering misdirected host defense mechanisms.

## Platelets, Polymorphisms, and Alloimmunization

Platelet component transfusions are extremely difficult to match for surface antigens between donors and recipients, apart from the ABO groups (A and/or B antigens can be variably expressed on platelets) (118). Moreover, platelets exhibit numerous copies of highly polymorphic HLA class I antigens. The functions associated with HLA class I molecules on platelets are currently under debate, as platelets are not consensually considered capable of presenting antigens. HLA transfer to other cells has recently been evidenced experimentally in mice, opening up novel avenues on the subject. HLA immunization of patients is not uncommon, but pre-storage leukoreduction has proven to be tremendously efficacious in limiting it, since leukocytes—10-times more loaded with HLA moieties than platelets—seem to potentiate immunization against platelet antigens, HLA, and human platelet antigens (HPA) (119). HPA are actually polymorphic variants of platelet glycoproteins, representing “platelet-specific blood groups.” Almost 20 such molecules are recognized as being immunogenic, with less than five being implicated in the most frequent immunization, while the others stand for rare antigens. Those HPA antigens usually come in two antithetical moieties termed “a” and “b,” “a” being the frequent allele and “b” the rarest. In certain circumstances, HLA or HPA testing and matching is the only option available to efficiently transfuse refractory patients; indeed, patients presenting with allogeneous anti-HLA and/or HPA Abs may destroy transfused platelets especially if Abs are directed at frequent Ags, leading to refractory states and imposing cross-matching of donor PCs against recipients’ plasma whenever possible (outside emergency situations) (120); rarely, transfer of allogenous Ags onto recipient’s platelets may create aggravated thrombocytopenic states with posttransfusion purpura (121). Transfusion of pooled platelets may be an option to saturate allo-Abs and give a chance to increase the patient’s platelet count during the critical phase (at the expense of creating further immunization, however). Regarding female patients having been alloimmunized during pregnancies, it is preferable to transfuse them using either HPA (HLA) typed, or cross-matched, PCs, to avoid the rebound of allo-Abs and refractoriness (122). It should be noted that as residual red blood cells exist even in very small numbers in PCs, patients transfused with PCs can be immunized against red blood cell antigens, especially when these are highly immunogenic such as Rhesus-D; this occurrence is nevertheless infrequent. Whereas it is strongly advised not to transfuse a Rhesus D negative female recipient in child-bearing age with platelets obtained from a Rhesus D positive donor unless prophylaxis is available if needed; Rhesus D negative men and females with no longer child-bearing potential are assumed to be safely transfused by Rh D positive donor’s PCs (especially pooled PCs according to recently published studies) (123); the case of Rhesus negative men (such as HSC transplanted) undergoing repeated PC transfusion is debated but should be discussed for Rh-D prophylaxis (124). Finally, it has recently been hypothesized that ABO mismatched platelets favor alloimmunization (125), although this hypothesis has yet to be ascertained with respect to its clinical impact.

## Conclusion

In conclusion, transfusion of platelets is generally safe and largely beneficial to patients. On rare occasions, SARs (which cannot be prevented by current measures), occur with clinical presentation of acute inflammation. In all cases investigated to date, either based on clinical observations or tested experimentally, BRMs (comprising chiefly of CKs and CHs and related molecules such as sCD40L) are found to be in close association. Potentially, these SARs are misdirected physiological defense mechanisms. This we have exemplified by the complex pathogenesis of HIT, which, however, involves just one of the many immunomodulatory CHs released by platelets. Additional safety measures to prevent those SARs would be beneficial to patients; however, it is likely they would be extremely difficult to establish and would not be cost effective. Again, transfusion-linked inflammation is likely the result of a combination of factors related to the donor, the BC, and the recipient. The only factor that can be targeted at present is the BC and measures to improve BC quality are being implemented when identified within the industry, in partnership with blood establishments. The identification of parameters that may be related to patients (recipients) would be desirable to identify at-risk patients and apply measures to prevent the severity of the hazards. If parameters are linked to donors, the situation becomes much more difficult, because further medical investigations in donors would scarcely be acceptable, and would have the potential to jeopardize BC stocks. How can one explain to a generous blood donor that he or she is perfectly safe and healthy, but “at risk” of inflicting harm on “certain” recipients? This problem is medically, ethically, and psychologically difficult to address. Alternatively, transfusion medicine may become one of the first medical specialties where personalized medicine comes into effect: “How can a given patient be given the BC most suited to his or her condition”?

## Author Contributions

CS, CA, HH-C, AG, OG, and FC: wrote the paper. CS, ST, CA, ChA, HH-C, PB, SL, AG, OG, and FC: participated in all steps of the process and reviewed the manuscript.

## Conflict of Interest Statement

The authors declare that the research was conducted in the absence of any commercial or financial relationships that could be construed as a potential conflict of interest.
